# Inducement of apoptosis by cucurbitacin E, a tetracyclic triterpenes, through death receptor 5 in human cervical cancer cell lines

**DOI:** 10.1038/cddiscovery.2017.14

**Published:** 2017-04-24

**Authors:** Ya-Min Cheng, Ching-Ju Shen, Chi-Chang Chang, Cheng-Yang Chou, Ching-Chou Tsai, Yi-Chiang Hsu

**Affiliations:** 1 Department of Obstetrics and Gynecology, National Cheng Kung University Hospital, College of Medicine, National Cheng Kung University, Tainan, Taiwan; 2 Department of Gynecology and Obstetrics, Kaohsiung Medical University Hospital, Kaohsiung Medical University, Kaohsiung, Taiwan; 3 Department of Obstetrics & Gynecology, E-Da Hospital, E-Da Hospital/I-Shou University, Kaohsiung, Taiwan; 4 Graduate Institute of Medical Science, College of Health Sciences, Chang Jung Christian University, Tainan, Taiwan; 5 Division of Gynecologic Oncology, Department of Obstetrics and Gynecology, Chang Gung Memorial Hospital and Chang Gung University College of Medicine, Kaohsiung, Taiwan; 6 Department of Obstetrics and Gynecology, Chang Gung Memorial Hospital, Chang Gung University College of Medicine Kaohsiung, Kaohsiung, Taiwan; 7 Bachelor Degree Program of Medical Sciences Industry, College of Health Sciences, Chang Jung Christian University, Tainan, Taiwan

## Abstract

Cervical cancer is the most common malignancy in women, for which conization or hysterectomy are the main therapy. Curcubitacin E (Cu E) is a natural compound-based drug which from the Guadi (climbing stem of *Cucumic melo L*). Previously shown to be an anti-tumor as well as a potent chemopreventive agent against several types of tumors. The present study, investigated anti-proliferation and apoptosis induced by Cu E in cervical cancer cell lines (HeLa and Ca Ski). The results indicate that the cytotoxicity is associated with accumulation in apoptosis but not necrosis. Cu E produced apoptosis as well as the up-regulation the expression of death receptor 5 (DR5). In addition, the DR5 gene activation in apoptosis, both effects increased proportionally with the dose of Cu E; however, mitosis delay was also dependant on the amount of Cu E treatment in the cancer cells. These results indicate that Cu E may delay cancer cell growth by apoptosis via upregulation of DR5 gene expression.

## Introduction

Cervical cancer is the sixth most common cancerous malignancy in females in Taiwan, and a leading cause of death among gynecological malignancies.^1^ The majority of women diagnosed with this cancer exhibit an advanced, widely disseminated malignancy and poor survival rate.^2^ Infections with human papillomavirus (HPV), predisposition, as well as various factors are believed to have important roles in the development of carcinogenesis.^3^ Overwhelming evidence has demonstrated that oncogenic types of HPV play an important role in the development of precursors of cervical cancer.^4^ However, only a small fraction of females infected with HPV develop the disease, indicating the contribution of other factors to the progression of lesions in invasive cervical cancer.^5^

Many studies indicate a positive correlation between the consumption of natural components of certain plants and the decreased incidence of some tumors including prostate, cervical, ovarian, lung and gastrointestinal tract.^6, 7^ Cucurbitacins are a group of tetracyclic triterpenes with medicinal properties derived from the climbing stem (Gua di) of *Cucumic melo L.*^8^ Gua di has been used extensively in traditional folk medicines throughout Asia, providing selective biological activities.^9^ Interest in this herb has grown in recent years due to its putative beneficial pharmacological effects as an anti-inflammatory^10^ and anti-cancer agent.^11^ There have also been indications that cucurbitacins may help to prevent and treat oxidative damage as well as suppress specific inflammatory factors.^12^

Cucurbitacin E (Cu E) is an active compound, which was previously shown to be a strong anti-feedant with the ability to disrupt cell actin and adhesion.^13^ Recent our studies have reported that Cu E has an inhibitory effect on the proliferation of cancer cells.^14^ However, it remains unclear whether Cu E inhibits the growth of cervical cancer cells. Furthermore, the mechanism underlying the anti-cancer effects of Cu E has yet to be identified.

This present study was initiated to investigate whether Cu E contributes to the anti-proliferation and apoptosis induction of cervical cancer cell lines (HeLa and Ca Ski). It is expected that these experiments will provide scientific basis and technological support for further development of cervical cancer therapy.

## Results

### Cu E can mediate the survival of cervical cancer cells, and thus inhibits their growth

**Figure 1:**
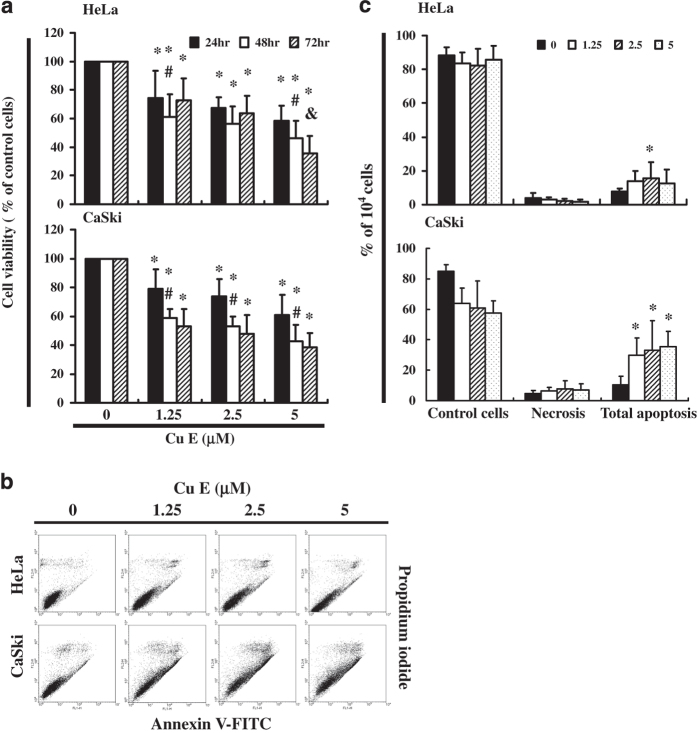
Cu E mediates the survival of cervical cancer cells (HeLa and Ca Ski), thereby inhibiting proliferation. (**a**) An *in vitro* study was initiated by treating each of the cell lines with increasing doses of Cu E (0, 1.25, 2.5 and 5 *μ*M) for 1–3 days. The survival of Cu E-treated cancer cell lines was then measured by MTT method. (**b**) Total apoptosis and necrosis in cervical cancer cell lines after 4 h of incubation with Cu E. (**c**) Results were expressed as a percentage of normal group (control cells), necrosis and the total number of apoptotic cells (early and late apoptosis). Results were expressed as a percentage of control, which was considered 100%. All data were reported as the mean (±S.E.M.) of at least three separate experiments. Statistical analysis was performed using a *t*-test, with significant differences determined at the level of *P<0.05 versus the control group, ^#^*P*<0.05 versus the 24 h group, ^&^*P*<0.05 *versus* the 48 h group.

To explore this anti-tumor activity, an *in vitro* study was conducted in which HeLa and Ca Ski cells were subjected to increasing dosages of Cu E (0, 1.25, 2.5 and 5 *μ*M) for 1 to 3 days. The proliferation of Cu E-treated cancer cells were then measured using the MTT method (Figure 1a). The results indicate that the survival and proliferation of Hela and Ca Ski cells was decreased by Cu E treatment in a dose- and time-dependent manner. (HeLa: *y*=−13.224*x*+108.09, *R*^2^=0.9077 (24 h), *y*=−16.585*x*+107.48, *R*^2^=0.8304 (48 h), *y*=−20.23*x*+118.58, *R*^2^=0.9672 (72 h); Ca Ski: *y*=−12.309*x*+109.15, *R*^2^=0.9432 (24 h), *y*=−17.794*x*+108.13, *R*^2^=0.8341 (48 h), *y*=−18.951*x*+107.19, *R*^2^=0.7949 (72 h)).

### Cu E-induced apoptosis of cervical cancer cell lines

To identify the role played by Cu E in the apoptosis/necrosis of cervical cancer cell lines, we employed Annexin V-FITC and propidium iodide staining to reveal the formation of apoptotic cells following 4 h of exposure to Cu E. The percentage of apoptotic cells was assessed by flow cytometric analysis (Figure 1b). A dot-plot of Annexin V-FITC fluorescence versus PI fluorescence indicates a significant increase in the percentage of apoptotic cells treated with Cu E, compared with untreated cells. Significant increase was observed in the percentage of cells undergoing apoptosis (Figure 1c).

### Cu E-induced accumulation of Sub G_1_ phase in Cu E-treated cells

**Figure 2:**
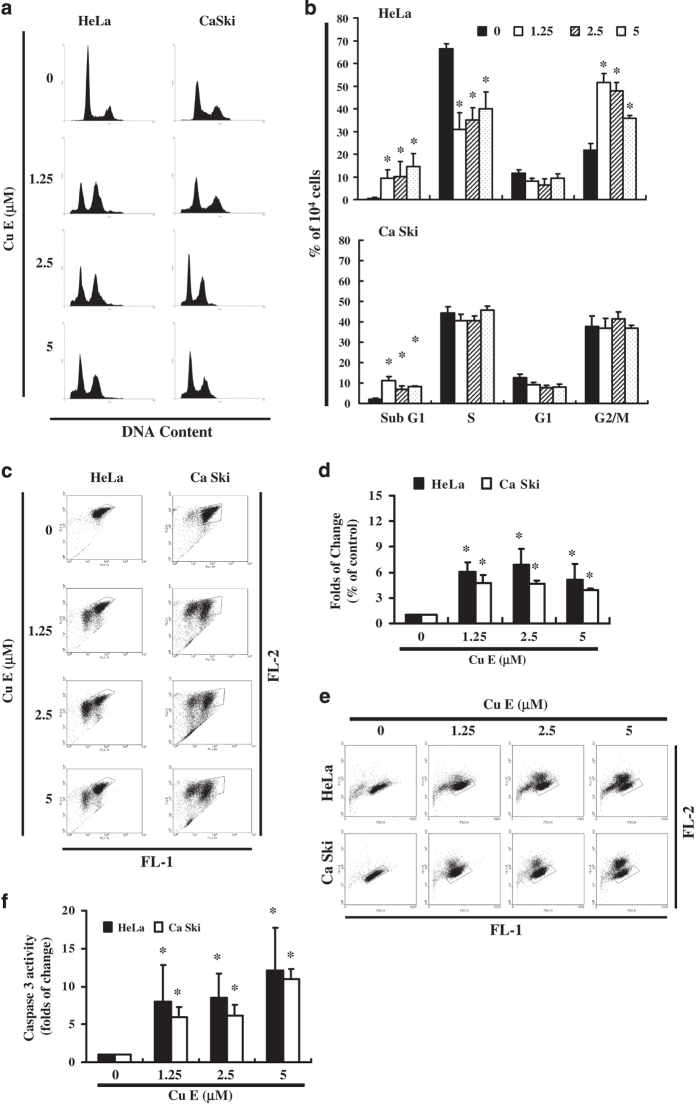
Influence of Cu E on cell cycle progression/distribution in cervical cancer cells: (**a**) Cell cycle analysis of HeLa and Ca Ski cells after being cultured with Cu E for 24 h. (**b**) Cu E induced an increase in sub G_1_ phase cells (%). (**c**) Cu E mediated mitochondrial membrane potential (ΔΨm)-dependent anti-proliferation in HeLa and Ca Ski cells. The levels of ΔΨm which was determined by JC-1 staining and flow cytometry and (**d**) Quantification through flow cytometry. (**e**) The cells were treated with Cu E for 24 h. Following treatment, the cells were harvested and labeled using FITC rabbit anti-active caspase 3. Activation was quantified by flow cytometry. (**f**) Quantification by flow cytometry. Symbol (*) in each group of bars indicates that the difference resulting from treatment with Cu E 0 *μ*M is statistically significant at *P*<0.05.

The cell cycle distribution of Cu E-treated cells was analyzed by flow cytometry. Cells were exposed to Cu E for 24 h before processing and analysis. As shown in Figure 2a, exposure to Cu E resulted in an increase in the number of G_2_/M phase and sub G_1_, cells, which may imply that the cervical cancer cells underwent cell cycle arrest and apoptosis. The results indicate that treatment with Cu E increased the cell populations in sub G1 (HeLa: *y*=2.5136*x*+3.1373, *R*^2^=0.8259; Ca Ski: *y*=0.7428*x*+5.4593, *R*^2^=0.1811), while simultaneously reducing the number of cells in the G_1_ phases in HeLa cells (*P<0.05 *versus* Cu E 0 *μ*M) (*y*=−3.6922*x*+51.15, *R*^2^=0.244) (Figure 2b).

### Assessment of changes in mitochondrial membrane potential

**Figure 3:**
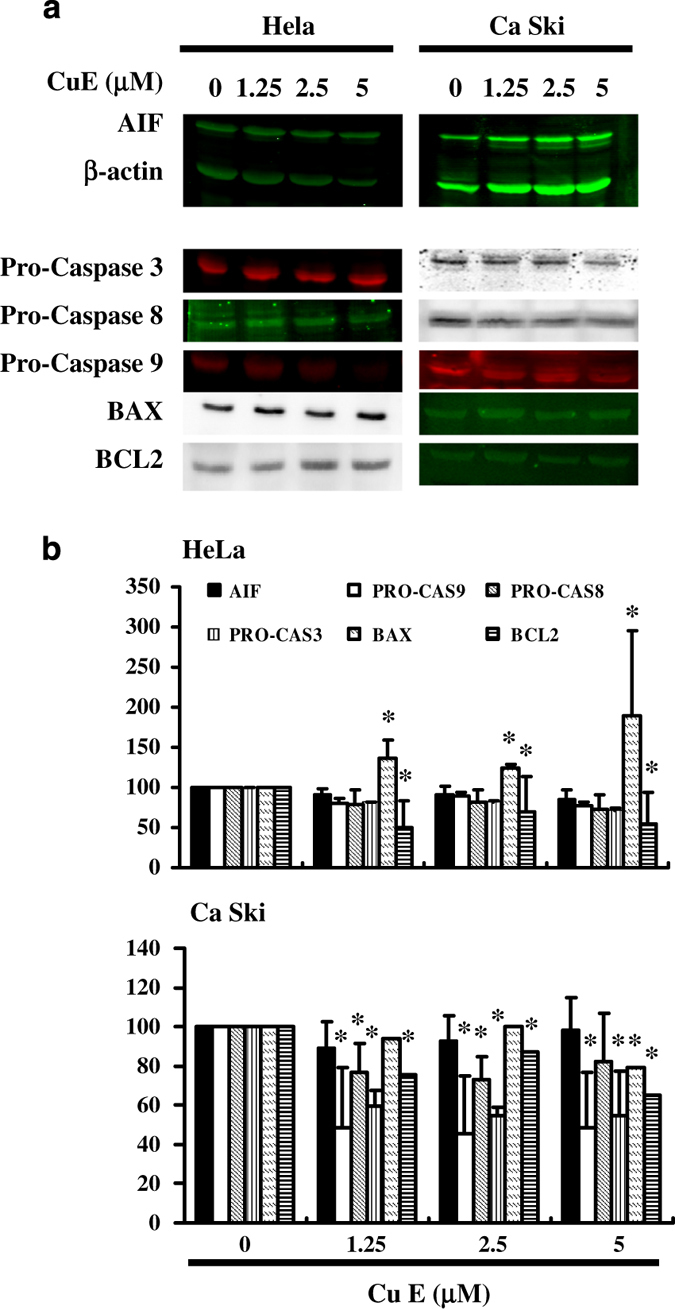
The genes down- or up-regulated in HeLa and Ca Ski cells following exposure to Cu E. The AIF, BAX, BCL-2 and caspase 3, −8, −9 gene expression profile was studied in cervical cancer cells exposed to the vehicle (DMSO) or to the Cu E. (**a**) Western blotting (**b**) qPCR. Symbol (*) in each group of bars indicates that the difference resulting from treatment with Cu E 0 *μ*M is statistically significant at *P*<0.05.

The loss of mitochondrial membrane potential is a hallmark for apoptosis. It is an early event coinciding with caspase activation. In non-apoptotic cells, JC-1 exists as a monomer in the cytosol (green) and accumulates as aggregates in the mitochondria, which appear red. In apoptotic and necrotic cells, JC-1 exists in monomeric form and stains the cytosol green. Figure 2c shows typical FL-1/FL-2 dot plots for JC-1 staining HeLa and Ca Ski cells with apoptosis. Cu E-free cancer cells are without apoptosis, which have red fluorescing J-aggregates. The green fluorescing monomers shown in the lower part indicate apoptotic cells. Figure 2d shows the percentages of apoptotic cells analyzed by flow cytometer in different Cu E-treated groups. Taken together, the observations imply that Cu E has significantly reduced the mitochondrial membrane potential of HeLa and Ca Ski cells. Moreover, we detected caspase 3 activation at Cu E concentrations of 1.25 to 5 *μ*M in HeLa (*y*=3.3754*x*−1.0423, *R*^2^=0.8835) and Ca Ski cells (*y*=1.9717*x*+3.0832, *R*^2^=0.8243) (Figures 2e and f), indicating induction of apoptosis in treated cells. There are also significant change was observed in the pro-caspase 3, -8 and -9 in Cu E-treated Ca Ski cells (Figures 3a and b).

In summary, the results summarized in Figures 1, 2, 3 suggest that Cu E affects the survival of cervical cancer cell lines inducing mitochondrial depolarization and apoptosis.

### Apoptosis induction in Cu E-treated cells via DR5 upregulation

**Figure 4:**
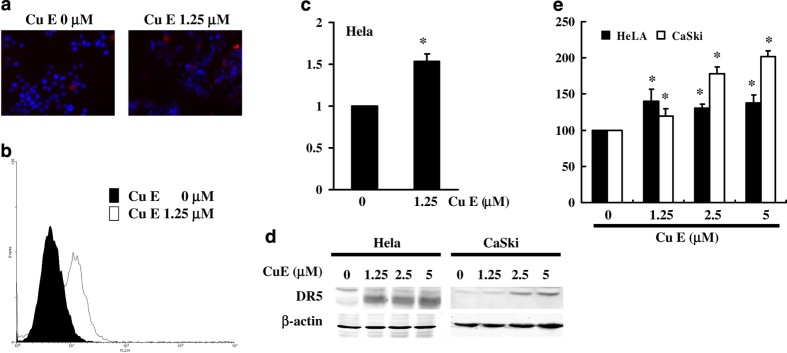
Influence of Cu E on DR5 expression in untreated and treated cancer cells. (**a**) Cells were dually stained using DAPI to analyze DNA content (blue) and DR5 protein (red) expression (**b**). (**c**) Quantification of DR5 expression was performed by flow cytometry following treatment with Cu E for 24 h. (**d**) Cu E enhanced the level of DR5 in HeLa and Ca Ski cells. (**e**) DR5 expression was subsequently detected using Western blot analysis: Representative blots from three independent experiments. Quantification of band intensities. Symbol (*) in each group of bars indicates that the difference resulting from treatment with Cu E 0 *μ*M is statistically significant at *P*<0.05. Scale bar: 10 *μ*m.

Figures 4a–c illustrate DR5 gene expression in cervical cancer cells (Figure 4a) and immunoblotting results of cellular proteins from HeLa and Ca Ski cell lines treated with Cu E (Figure 4d). Gene expression and western blot analysis revealed an increase in DR5 following incubation with Cu E (Figures 4d and e).

These data suggest that DR5 level regulated the tumorigenicity of cervical cancer cells via Cu E. These findings indicate that common molecular pathways are involved in inducing apoptosis. Findings from qPCR analysis were further validated by a microarray analysis (data not shown), which indicated substantial TNF up-regulation (relative expression ratio 3.08) as well as notable upregulation of DR5 (relative expression ratio 7.46) in cervical cancer cells following exposure to Cu E.

## Discussion

Cu E or *α*-elaterin is a natural compound previously shown to be an anti-feedant drig as well as a potent chemotherapy agent against several types of tumor.^15^ The most common cell death mode on Cu E treatment seems to be apoptosis, cell cycle regulation and many studies have attempted to elucidate the mechanism underlying the anti-cancer activity of this compound.^16^

Tumor necrosis factor (TNF)-related apoptosis-inducing ligand (TRAIL) is a secreted protein belonging to tumor necrosis factor superfamily of cell death-inducing ligands.^17^ TRAIL drives the onset of extrinsic apoptosis pathways after binding to its receptors Death receptor 4 (DR4, Trail-R1, Apo2 and Tnfrsf10A) and Death receptor 5 (DR5, Trail-R2, Trick2 and Tnfrsf10B).^18^ DR5 is a cell surface receptor of the TNF-receptor superfamily that binds TRAIL and mediates apoptosis.^19^ it is a promising target for cancer therapy due to its ability to selectively induce apoptosis in cancer cells.^20,21^

In this study, Cu E demonstrated anti-proliferation activity as well as the ability to induce apoptosis. We found that treating cervical cancer cells with Cu E resulted not only the repressed the progression of cell cycle in G_2_/M (Cu E 1.25 and 2.5 *μ*M; Figures 2a and b) but also induced the apoptosis by upregulation of DR5.

The results collected in this series of studies provide experimental evidence supporting the contention that Cu E may irreversibly arrest the growth of cervical cancer cells. The results of mechanistic analysis led to the conclusion that both the inhibition of proliferation and the induction of apoptosis are highly dependent upon Cu E accumulated in the cancer cells.

In conclusion, this study demonstrates for the first time that Cu E is an effective inhibitor of cervical cancer. The role of Cu E in the inhibition of tumor growth was highlighted by apoptosis induction through the upregulation of DR5 expression. These findings suggest the applicability of Cu E as an anti-tumor agent for cervical cancer chemoprevention.

## Materials and methods

### Materials

Cucurbitacin E, DMSO (dimethyl sulfoxide) and MTT [3-(4,5-dimethylthiazol-2-yl)- 2,5-diphenyltetrazolium bromide] were obtained from Sigma-Aldrich (St Louis, MO, USA). Cell culture medium (MEM and RPMI 1640), phosphate-buffered saline (PBS), anti-biotics, sodium pyruvate, l-glutamine, trypsin and fetal bovine serum (FBS) were purchased from Gibco, BRL (Grand Island, NY). Polyvinylidene fluoride membrane (PVDF) (Millipore), and molecular weight marker were purchased from Bio Rad (USA). All other reagents and compounds were analytical grades.

### Cells

The HeLa and Ca Sk cells were purchased from ATCC. The cells were maintained on culture dishes, in 90 % (v/v) MEM Eagle or RPMI1640 with 2 mM l-glutamine and Earle’s BSS adjusted to contain 1.5 g/l sodium bicarbonate, 0.1 mM non-essential amino acid (NEAA) and 1 mM sodium pyruvate with 10% (v/v) FBS. The cells were cultured in an atmosphere containing 5% CO_2_ at 37 °C incubator.

### Cell proliferation assay

The cancer cells were seeded into 96-wells culture plate at 5000 cells/well. The cells were treated with 0, 1.25, 2.5 and 5 *μ*M Cu E, the Cu E will complex with culture medium. Then the cell will incubate in 37 degree C for 24, 48 and 72 h in the CO2 incubator. After incubate 24, 48 and 72 h. The cell treatments MTT dye (1 mg/ml) at least 4 h on each well. The reaction was stopped by the addition of DMSO, and optical density was measured at OD_540_ on a multi-well plate reader. Background absorbance of the medium in the absence of cells was subtracted. All samples were assayed in triplicate, and the mean for each experiment was calculated. Results were expressed as a percentage of control, which was considered as 100%. Each assay was carried out in triplicate and the results were expressed as the mean (±S.E.M.).

### Measurement of apoptosis/necrosis

Cervical cancer cell lines were first seeded in 6-well plates (Orange Scientific, E.U.). Following treatment with Cu E for 4 hours, the cells were harvested. The cells were re-centrifuged (the supernatant discarded) and resuspended/incubated in 1X annexin-binding buffer. Five microliter of annexin V-FITC (BD Pharmingen, Franklin Lakes, NJ, USA) and 1 *μ*l of 100 *μ*g/ml PI working solution for 15 min. Following the incubation period, the stained cells were analyzed using flow cytometry (FACSCalibur, BD, San Jose, CA, USA). Data was analyzed using WinMDI 2.9 free software (BD).

### Caspase 3 activity assay

The caspase activity was assessed by the FITC rabbit anti-active caspase 3 (BD Pharmingen). The cells were treated with Cu E for 24 h. Caspase 3 activition level was then measured by the flow cytometry (FACSCalibur). Data was analyzed using WinMDI 2.9 free software (BD)

### Mitochondrial membrane potential

The cell lines were first seeded in 6-well plates (Orange Scientific). Following treatment with Cu E for four hours, JC-1 (25 *μ*M) was added to the culture medium (500 *μ*l/well) and incubated (37 °C, 20 min) for mitochondrial staining. After washing twice with warm PBS, the cells were fixed using 2% paraformaldehyde. For JC-1, quantification by flow cytometry (BD FACScalibur) and mitochondria containing red JC-1 aggregates in healthy cells were detectable in the FL-2 channel, and green JC-1 monomers in apoptotic cells were detectable in the FL-1 channel.

### Cell cycle analysis

For cell cycle analysis we used the fluorescent nucleic acid dye propidium iodide (PI) to identify the proportion of cells in each of the three interphase stages of the cell cycle. The cells were treated with Cu E for 24 h, and then harvested and fixed in 1 ml cold 75% ethanol for over night at −20 °C. DNA was stained in PI/RNaseA solution and the DNA content was detected using flow cytometry. Data was analyzed using WinMDI 2.9 free software.

### DR5 expression

Confocal microscopy was performed as described previously.^22^ Briefly, The cells (2×10^6^ cells) were fixed on coverslips. After treatment, they were incubated with mouse anti-DR5-phycoerythrin antibody (sc-166624 PE) (Santa Cruz BioTechnology, Dallas, TX, USA) for 30 min, and then washed with PBS. The cells were mounted onto microscope slides using mounting medium containing DAPI. The cells were first seeded in 6-well plates (Orange Scientific). Following treatment and anti-DR5-PE antibody incubated (37 °C, 20 min) for protein staining. After washing twice with PBS, the cells were fixed using 2% paraformaldehyde. For DR5, quantification by flow cytometry (BD FACScalibur).

### Western blot assay

A total of 50–100 *μ*g of proteins were separated by 10–12% SDS-PAGE, and transferred to PVDF membranes (Millipore, USA). The membranes were blocked with blocking buffer (Odyddey, USA) overnight, and incubated with anti-*β*-actin (Sigma-Aldrich), anti-caspase 3 (sc-7148), anti-caspase 8 (sc-6134), anti-caspase 9 (sc-7885), anti-AIF (sc-9417) and DR5 (sc-7192) antibodies for 1.5–2 h. The blots were washed and incubated with a second antibody (IRDye Li-COR, USA) or conjugated with horseradish peroxidase (HRP) at a 1/20,000 dilution for 30 min. The antigen was then visualized using a near infrared imaging system (Odyssey LI-COR, Lincoln, NE, USA) or chemiluminescence detection kit (ECL; Amersham Corp., Arlington Heights, IL, USA). The data was analyzed using Odyssey 2.1 software.

### RT-PCR

A reverse transcriptase system (Promega, Southampon, UK) was used to synthesize cDNA from 1 *μ*g of total RNA. Between 2 and 4 *μ*l of cDNA were used for PCR analysis. PCR (50 *μ*l) reactions were performed using 100 ng of each primer and 1 unit of Dynazyme II (Flowgen, Lichfield, UK). Thermal cycling was conducted for 35 cycles at the following temperature/durations: 98 °C for 10 s, 66 °C for 30 s, and 72 °C for 1 min using a Progene thermal cycler (Cambridge, UK). A final extension of 72 °C was performed for 10 min at the end of 35 cycles. The primers used for amplification of the target genes were checked against all other gene sequences for specificity. PCR reactions were analyzed on 1.5% agarose/TAE minigels and stained using 0.5 *μ*g/ml ethidium bromide. Gels were visualized using an Apligene UV CCD camera system.

### Real-time PCR

Real-time PCR was conducted using SYBR Green PCR MasterMix according to the manufacturer’s instructions. Quantitative real-time PCR (qRT-PCR) was performed using approximately 200 ng of SYBR Green PCR MasterMix in an ABI 7300 system (Applied Biosystems, Foster City, CA, USA). PCR conditions were 95 °C for 120 s, 60 °C for 30 s, and 72 °C for 30 s for 40 cycles. Sample cells from three plates were run in duplicate, using the threshold suggested by the software for the instrument to calculate Ct. To normalize readings, we used Ct values from 18s as internal controls for each run, obtaining a delta Ct value for each gene. (AIF F: GATTGCAACAGGAGGTACTCCAAGA, R: GATTTGACTTCCCGTGAAATCTTCTC; Caspase 3 F: GGAAGCGAATCAATGGACTCTGG R: GCATCGACATCTGTACCAGACC; Caspase 8 F: AGAAGAGGGTCATCCTGGGAGA R: TCAGGACTTCCTTCAAGGCTGC; Caspase 9 F: TTCCCAGGTTTTGTTTCCTG, R: CCTTTCACCGAAACAGCATT; BAX F: TCAGGATGCGGTCCACCAAGAAG, R: TGTGTCCACGGCGGCAATCATC; BCL-2 F: GCCACTTACCTGAATGACCACC, R: AACCAGCGGTTGAAGCGTTCCT; and GAPDH F: GTCTCCTCTGACTTCAACAGCG R: ACCACCCTGTTGCTGTAGCCAA).

### Statistical analysis

All data were reported as the mean (±S.E.M.) of at least three separate experiments. A *t*-test or one-way ANOVA with *post hoc* test was employed for statistical analysis, with significant differences determined as *P*<0.05.
